# Impact of COVID-19 on medical students’ mental wellbeing in Jordan

**DOI:** 10.1371/journal.pone.0253295

**Published:** 2021-06-17

**Authors:** Khaled Seetan, Mohammad Al-Zubi, Yousef Rubbai, Mohammad Athamneh, Almu’atasim Khamees, Tala Radaideh

**Affiliations:** 1 Department of Clinical Sciences, Faculty of Medicine, Yarmouk University, Irbid, Jordan; 2 Princess Aisha Bint Al-Hussein College of Nursing and Health Sciences, Al-Hussein Bin Talal University, Maan, Jordan; 3 Faculty of Medicine, Yarmouk University, Irbid, Jordan; Medical University of Vienna, AUSTRIA

## Abstract

COVID-19 has spread throughout the world and has resulted in significant morbidity, mortality, and negative psychological impact. This prospective cross-sectional study is exploring the effect of the pandemic on mental health of medical students. The study was conducted at six Jordanian medical schools using an online survey to collect students’ socio-demographic and academic data. Assessment of mental wellbeing status was done using Kessler’s psychological stress scale (K10); the impact of COVID-19 on life activities and strategies followed to manage the situation were also examined. A total of 553 medical students were recruited for the study. Men constituted 40.1%, and women were 59.9%. Students reported that COVID-19 has affected the aspects of physical fitness (73.1%), study (68.4%), and social relationships (65.6%) the most. Sixty-six percent of the students were concerned about family members’ affection, and more than half (58.4%) explained their concerns about the inability to get clinical sessions and labs. Cooking, baking, and hobby practicing were the most popular methods to improve their mental wellbeing. About half of the participants had a severe mental disorder, and only 13.2% were likely to be well. The study indicates that half of our medical students suffer severe mental disorders, with physical fitness, exercise, and studying being among the most affected aspects during the COVID 19 pandemic. It is recommended that measures need be taken to alleviate students’ stress, which might have deleterious effects in many aspects.

## Introduction

Since the reported cluster of patients with fever, cough, and pneumonia symptoms in Wuhan—China, the coronavirus (SARS-COV-2) has spread worldwide to affect more than 2 million people. The disease was then declared a pandemic by the World Health Organization (WHO) [1, 2]. Due to its highly contagious nature and the rising toll of mortality among the general population, many countries have locked down and quarantined their population to control the spread of the disease. Such restrictive measures were showing effectiveness although many concerns have emerged regarding their psychological impact and their effect on the mental wellbeing of the general population, especially those with high vulnerability to mental health diseases. Like mental health, diet behavior has also been affected by the lockdown as evident by a web based survey that found higher rates of eating and snacking resulting in pronounced weight gain, especially among already overweight and older subjects Moreover, higher rate of intimate partners violence, alcohol consumption and cigarette smoking was reported compared to the time before lockdown [3–7].

Until the 24th of April 2021, a total of 699,164 confirmed Covid-19 cases with 8,514 deaths were reported in Jordan [8]. The first reported case of Covid-19 was detected on the third of March 2020, after which the number of cases increased dramatically to become 89 cases by March 15. Eventually, the number of cases continued to rise.

In attempts to control the situation, local authorities have introduced public health infection prevention and control measures such as social distancing, seizing all inbound, outbound, and international travels besides eliciting minister of defense as an authorized body to formulate orders accordingly [9].

Subsequently, a total lockdown on all borders arriving from pandemic countries was put into effect before 17th of March and isolation of administrative governorates from each other was done. In addition, social media, ministry of health and nongovernmental organizations played a crucial role by promoting public health awareness about the pandemic. Only medical and nursing staff, armed forces were allowed to move freely during the curfew time. As a result, studying in all educational institutions including universities was also banned at that time [9, 10].

During the COVID-19 pandemic, there was an increased prevalence of anxiety, fear, depression, sleep disturbance, somatization, and OCD disorders among health care workers, which were especially prevalent among individuals working in high-risk units such as emergency unit and ICU [11–13].

Different pandemic related stressors have been identified, such as the unpredictable course of infections [14], lack of transparency form authorities [15], personal restrictions and sudden changes, the inability of future planning, constant concerns about the health of individuals and their relatives [16], and economic impact and financial losses [17]. A recent meta-analysis showed that the prevalence of anxiety among the general population during the period of the COVID-19 pandemic was 31.9%, which supports those observations [18].

In general, medical students are known to have higher levels of psychological impairment in the form of depression, anxiety, and stress than the general population; this is due to the high-pressure environment where they study and train. In turn, COVID-19 is considered an additional source of stress to the general population and especially to healthcare workers and medical students [19–21].

Awareness about the medical community’s mental health concept was difficult to achieve even before the lockdown due to the COVID-19 pandemic. There have been many published reports that attempted to address the problem of psychological impact on medical students. Since no adequate data was available regarding the mental health status among medical students in Jordan, this study aimed to determine the existence of, and gauge the negative impact of COVID-19 on mental health wellbeing of medical students.

## Materials and methods

A cross-sectional study was conducted among medical students in Jordan during the period from October to December 2020. The study utilized an online questionnaire which was prepared and administered through Google platforms. The questionnaire inquired about the study participants’ sociodemographic characteristics (age, gender, college attended, and academic year) and a self-rated assessment of the effect of COVID-19 on the mental wellbeing status using the Kessler psychological stress scale 10 (K10). K10 involves ten questions with a five-level response scale; each of them is scored from one "none of the time" to five "all of the time." Scores of the ten questions were then summed with a minimum score of 10 and a maximum of 50. Low scores indicate low levels of psychological stress, while high scores indicate high psychological stress levels [22]. Other questions targeted the impact of COVID-19 on various areas of life, concerns about COVID-19, and activities and strategies followed by the participants to manage the situation.

A sample size of 575 students was estimated using Epiinfo 7 stat calculator software, assuming a prevalence of 68% [23], precision of 0.04, 95% confidence interval, and a 10% non-response rate. All medical students were eligible to participate. Informed written consent was taken from each student, and participation was voluntary, with no identifying data collected from the participants. This study has been conducted in accordance with the Helsinki declaration. The research design and ethical considerations were reviewed and approved by the Institutional Review Board at King Abdullah University Hospital.

Data were then collected using a Google form, with organization and statistical analysis performed using statistical package of social sciences (SPSS) version 25. Categorical variables were described by frequency distribution, while the mean and standard deviation described continuous variables. Independent samples t-tests, and one-way ANOVA were used to compare mean psychological stress scores between groups. A p-value of ≤ 0.05 was considered as a cutoff for statistical significance for all pruposes.

## Results

A total of 553 medical students participated in the study. The mean age was 20.7 years (SD = 1.9), with women (59.9%) constituting the majority of study participants. The students of Yarmouk University represented the bulk of the study population with 209 students (37.8%), while other schools contributed with varying proportions. Nearly half of the students were third-year students (23.1%) and second-year students (22.4%). The remaining half of the sample were relatively evenly distributed amongst first, fourth, and sixth year students (Table 1).

**Table 1:** Demographic characteristics of the study participants.

		Mean	SD	N	N %
Age		20.7	1.9		
Are you a medical student in Jordan?	Yes			553	100.0%
	No			0	0.0%
Gender	Male			222	40.1%
	Female			331	59.9%
Medical college	AL-Balqa Applied University			50	9.0%
	Hashemite University			72	13.0%
	Jordan University			97	17.5%
	Jordan University of Science and Technology			71	12.8%
	Mutah University			54	9.8%
	Yarmouk University			209	37.8%
Year of study	First Year			59	10.7%
	Second Year			124	22.4%
	Third Year			128	23.1%
	Fourth Year			70	12.7%
	Fifth Year			83	15.0%
	Sixth Year			89	16.1%

**Fig 1:**
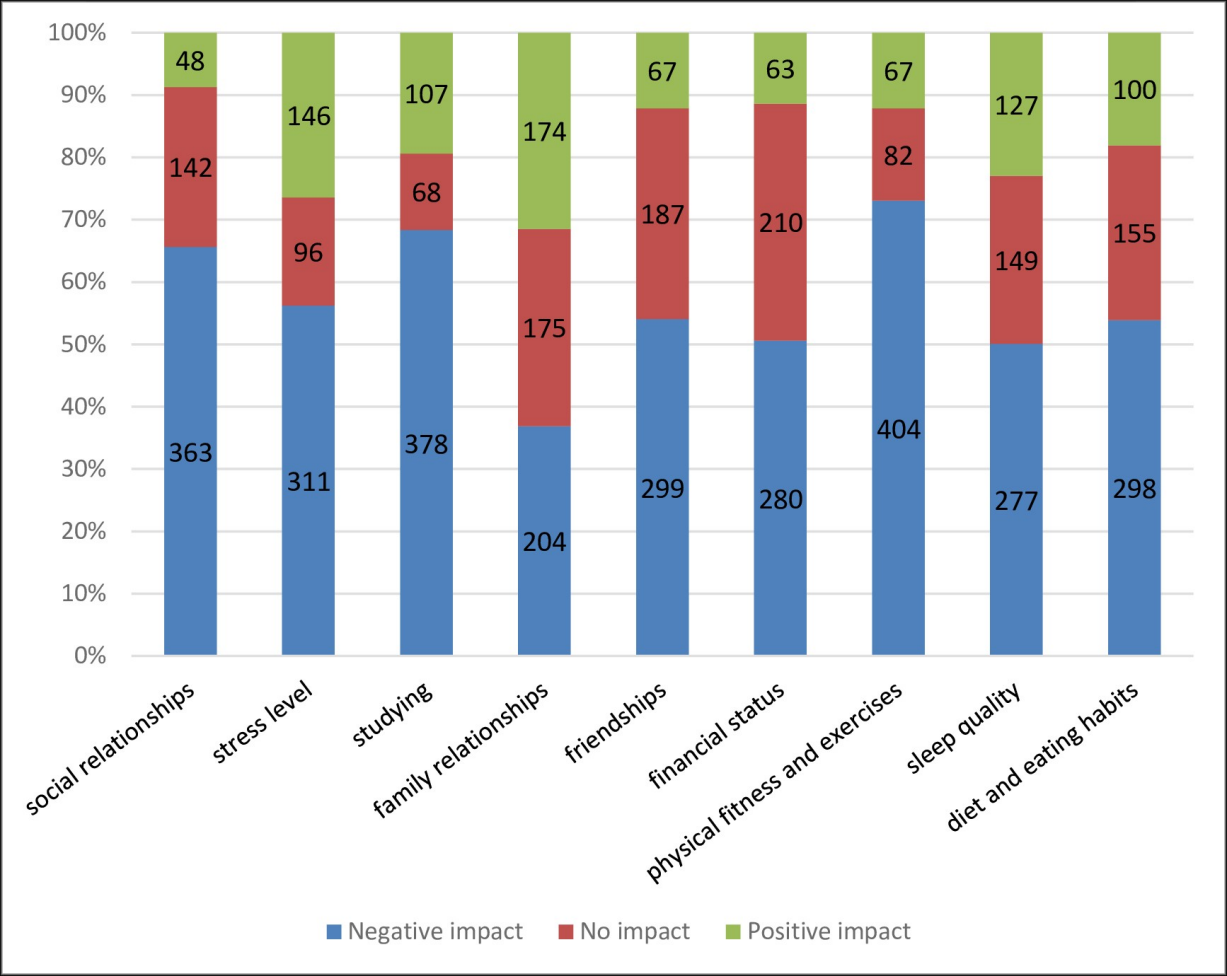
The impact of Covid-19 on different aspects of medical students’ lives.

More than half of students (65.6%) claimed that their social relationships had been negatively impacted, while 56.2% have had a negative impact on stress level, and about two-thirds of the participants said that COVID-19 affected their studying (68.4%). COVID-19 negatively affected friendships, and familial relationships in 54.1, and 36.9% of the students, respectively. Nearly half of the participants (50.6%) have been affected regarding the financial aspects. COVID-19 has affected the physical fitness and exercise habits of 73.1% of students, and about half of them (50.1% and 53.9%) claimed that it had a negative effect on sleep quality and eating habits, respectively (Fig 1).

Most students expressed concerns about contracting the disease, and having family members infected. Additionally, concerns about academic influence were raised by the students as more than half of the students (58.4%) could not acquire more clinical skills and attend labs, while 41.8% were afraid of being self-isolated for an extended period. Moreover, limitations on attending course classes and exams were noted by more than one-fifth of the students, either due to difficulties in using technologies, or restriction of travel. Nearly a third of the participants were concerned about getting a suitable residency program (35.4%), while 23.1% displayed concerns about being able to graduate in the first place, and 17.9% were concerned about being withdrawn from medical school (Table 2).

**Table 2:** Concerns of participants about negative impact of COVID-19.

Issues of concern regarding the negative impacts of COVID-19 on personal and academic life	N	N %
I have concerns about being infected by COVID 19	180	32.5%
I have concerns about being unable to acquire more clinical skills and being not able to attend labs	323	58.4%
I have concerns about being self-isolated for long time	231	41.8%
I have concerns about being unable to use new technologies to attend online classes and seminars	115	20.8%
I have concerns about withdrawing from medical school	99	17.9%
I have concerns about being unable to find a suitable residency training post after graduation	196	35.4%
I have concerns about being unable to get graduated from medical school	128	23.1%
I have concerns about being unable to travel abroad to do exams or attend elective courses	170	30.7%
I have concerns about having one of my family member being infected with COVID 19	365	66.0%

When asked about measures used to improve mental wellbeing, most of the participants chose practicing hobbies like playing and listening, and cooking and baking (68.9% for both), this is followed by praying and meditation (60% for both), other choices such as video chats and social media apps, seeking psychotherapy advice, and learning a new language were less popular measures (Table 3).

**Table 3:** Activities and measures used by the participants to improve mental wellbeing.

Which of the following activities and measures you used to improve your mental well being	N	N %
Video chats and social media apps	251	45%
Seeing a psychotherapy specialist	79	14.3%
Cooking and baking	381	68.9%
Practicing hobbies like playing and listening to music	381	68.9%
Praying	332	60.0%
Meditation	332	60.0%
Learning new language	86	15.6%
Exercise and fitness	299	54.1%

Table 4 shows participants’ responses to the K10 scale questions. Overall, the mean psychological distress score of students was 29.6 ± 8.74, with scores categorized into low, moderate, high psychological distress. Nearly half of the students were likely to have a severe disorder (50.3%) according to psychological stress score, while 20.1% were likely to have a moderate disorder, and 16.5% with a mild disorder, while only 13.2% were likely to be well (Table 5). Moreover, there were statistically significant differences in psychological distress scores with different students’ genders (p < 0.001), age (p = 0.001), and year of study (p < 0.001) (Table 6).

**Table 4:** Responses of participants to the (K10) scale questions.

	None of the time		A little of the time		Some of the time		Most of the time		All of the time	
	N	N %	N	N %	N	N %	N	N %	N	N %
In the past 4 weeks, about how often did you feel tired out for no good reason?	32	5.8%	86	15.6%	186	33.6%	194	35.1%	55	9.9%
In the past 4 weeks, about how often did you feel nervous?	23	4.2%	90	16.3%	188	34.0%	174	31.5%	78	14.1%
In the past 4 weeks, about how often did you feel so nervous that nothing could calm you down?	148	26.8%	144	26.0%	144	26.0%	92	16.6%	25	4.5%
In the past 4 weeks, how often did you feel hopeless?	78	14.1%	111	20.1%	158	28.6%	150	27.1%	56	10.1%
In the past 4 weeks, about how often did you feel restless or fidgety?	48	8.7%	114	20.6%	189	34.2%	147	26.6%	55	9.9%
In the past 4 weeks, about how often did you feel so restless you could not sit still?	130	23.5%	153	27.7%	151	27.3%	90	16.3%	29	5.2%
In the past 4 weeks, about how often did you feel depressed?	48	8.7%	125	22.6%	157	28.4%	140	25.3%	83	15.0%
In the past 4 weeks, about how often did you feel that everything was an effort?	39	7.1%	103	18.6%	155	28.0%	165	29.8%	91	16.5%
In the past 4 weeks, about how often did you feel so sad that nothing could cheer you up?	92	16.6%	133	24.1%	160	28.9%	120	21.7%	48	8.7%
In the past 4 weeks, about how often did you feel worthless?	148	26.8%	116	21.0%	131	23.7%	102	18.4%	56	10.1%

**Table 5:** Distribution of levels of mental disorders of the participants.

		Mean	SD	N	N %
Psychological distress score		29.60	8.74		
Likelihood of having a mental disorder	Likely to be well			73	13.2%
	Likely to have a mild disorder			91	16.5%
	Likely to have a moderate disorder			111	20.1%
	Likely to have a severe disorder			278	50.3%

**Table 6:** Comparison of psychological distress scores among different groups of students.

		Psychological distress score		*p* (F/t)
		Mean	Standard Deviation	
Age groups	≤ 20	30.93	8.22	0.001* (7.05)
	21–23	28.14	9.13	
	≥ 24	28.48	8.69	
Gender	Male	27.46	8.89	< 0.001* (-4.81)
	Female	31.04	8.36	
Medical college	AL-Balqa Applied University	29.84	9.30	0.805 (0.46)
	Hashemite University	29.14	8.23	
	Jordan University	29.33	8.47	
	Jordan University of Science and Technology	29.25	9.18	
	Mutah University	31.22	9.66	
	Yarmouk University	29.53	8.56	
Year of study	First Year	31.15	8.80	< 0.001* (4.96)
	Second Year	32.33	8.12	
	Third Year	29.30	8.00	
	Fourth Year	28.57	9.24	
	Fifth Year	28.46	9.27	
	Sixth Year	27.07	8.73	

* Indicate statistical significance.

## Discussion

This study meant to address the impact of COVID-19 on mental health and wellbeing among medical students in Jordan. As the pandemic has resulted in significant mental health impairment that affected even the general population worldwide, the disease has left more pressure on health care professionals [24]. As public health emergencies have many psychological effects, the levels of anxiety, depression, and other mental health problems have been worryingly rising since the declaration of COVID-19 as a pandemic. While these debilitating psychological conditions might have resulted from lifestyle modifications and restricting decisions from higher authorities [24, 25], the effect of the disease on medical students is expected to be higher since they are a more vulnerable group and at risk of exposure more than the general population [20]. The majority of our medical students (73.1%) have found it difficult to attend physical fitness sessions and exercise, similar to previously reported findings [26, 27]. Unfortunately, this activity, along with peer activities, were the most effective non-pharmacological therapy among college and university levels to alleviate negative emotions [3, 28]. This difficulty facing the students to access and conduct such sessions may directly contribute to such high anxiety and stress levels.

A significant impact that may possess long term consequences and affect the medical students’ career is the pandemic’s impact on the study. This effect may result from the direct psychological effect of the pandemic. Additionally, some students may find it challenging to adapt to the new teaching methods, such as online classes and online meeting apps; they may start to feel lacking behind compared to their colleagues, which is an additional anxiety and stress source.

Having affected family members was the most concerning scenario for our medical students (66%), which is quite similar to that reported (62%) by Lyons et al. [23]. This percentage was much higher in a similar study where almost all of the student participants were afraid of such a situation [20]; this fear may be because the disease is not well understood, and hard to anticipate its course besides the high reported number of mortality among the elderly population. Also, the financial impact of the disease is an important issue to be considered, as the economic status at the country and individual levels were found to be significantly impacted due to the outbreak [29]; this resulted in loss or reduction of sources of income and students may feel anxious and stressed about paying their tuition and courses’ fees.

Practicing hobbies and cooking were the most strategies selected to alleviate stress, while another Australian study concluded that most of the students used video chats and social media apps [23]. Severe disorder according to psychological stress score was accounted for half of our study population, in contrast with Australian study where only 11% and far more than the reported 7.3% in another study [20, 23]; this is a significantly alarming finding given the low reported rates in other studies’ findings.

This study shows an increasing magnitude of stress and mental wellbeing disorders, which contradicts previous findings reporting lower anxiety levels among medical students than their non-medical peers, although medical students are expected to have a better perception and sufficient information about COVID-19 transmission, course, and prognosis. As medical students received information from reliable sources, this in turn, should contribute to a reduced level of anxiety and fear [30].

In the current time, aims to improve students’ mental wellbeing are essential issues. It is becoming a significant priority to protect them from the debilitating consequences of mental disorders and reduce anticipated negative effects of the pandemic.

## Study limitations

The objective responses of the participants to the survey questioners based on their perceptions and COVID 19 experiences make it difficult to rely on the collected data to reach a diagnosis of mental illnesses. Depression and anxiety disorders diagnosis need direct individual clinical assessment. Another issue is that the data shows the mental well-being status of participants at certain point of time and does not follow the progression of their anxiety and depressive symptoms. Also it is expected that individuals with severe COVID 19 negative mental impact did actively participate in the survey as those individuals may had mental difficulties which makes them under presented in our study. Additionally, due to the setting of data collection, volunteer and response bias may have arisen.

## Conclusion

The study has provided insight into the impact of the COVID-19 pandemic on mental wellbeing and highlighted the major coping strategies followed by medical students to control the situation. We conclude that half of our medical students suffer severe mental disorders, with physical fitness, exercise, and studying are the most affected aspects during the pandemic. The affection of family members was the primary issue concerning students. It is recommended that measures be taken to alleviate students’ stress, which might have deleterious effects in many aspects.

## Supporting information

S1 Dataset (XLSX)
